# The Role of Circulating Biomarkers in Lung Cancer

**DOI:** 10.3389/fonc.2021.801269

**Published:** 2022-01-21

**Authors:** Sayuri Herath, Habib Sadeghi Rad, Payar Radfar, Rahul Ladwa, Majid Warkiani, Ken O’Byrne, Arutha Kulasinghe

**Affiliations:** ^1^ Department of Medical Laboratory Sciences, Faculty of Health Sciences, The Open University of Sri Lanka, Nugegoda, Sri Lanka; ^2^ Centre for Genomics and Personalised Health, Faculty of Health, School of Biomedical Sciences, Queensland University of Technology, Brisbane, QLD, Australia; ^3^ Faculty of Engineering and IT, School of Biomedical Engineering, University of Technology Sydney, Sydney, NSW, Australia; ^4^ Princess Alexandra Hospital, Cancer Care Services, Woolloongabba, QLD, Australia; ^5^ The University of Queensland Diamantina Institute, The University of Queensland, Brisbane, QLD, Australia

**Keywords:** Circulating tumour cells, cell-free DNA, circulating tumour DNA, microRNA and exosomes, lung cancer, liquid biopsy

## Abstract

Lung cancer is the leading cause of cancer morbidity and mortality worldwide and early diagnosis is crucial for the management and treatment of this disease. Non-invasive means of determining tumour information is an appealing diagnostic approach for lung cancers as often accessing and removing tumour tissue can be a limiting factor. In recent years, liquid biopsies have been developed to explore potential circulating tumour biomarkers which are considered reliable surrogates for understanding tumour biology in a non-invasive manner. Most common components assessed in liquid biopsy include circulating tumour cells (CTCs), cell-free DNA (cfDNA), circulating tumour DNA (ctDNA), microRNA and exosomes. This review explores the clinical use of circulating tumour biomarkers found in liquid biopsy for screening, early diagnosis and prognostication of lung cancer patients.

**Graphical Abstract:**
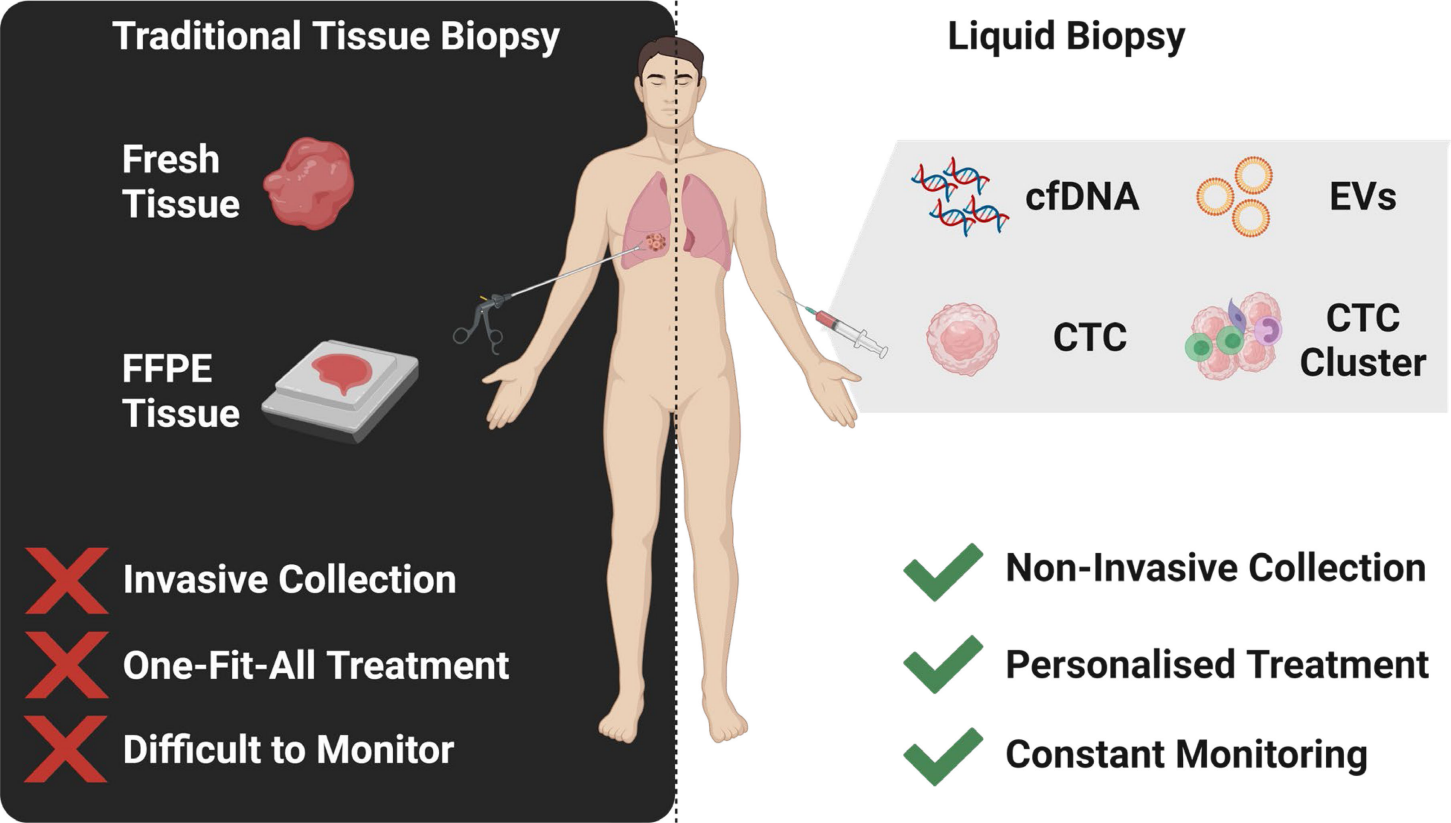
The identification of multi-marker analytes in liquid biopsy samples enables a personalised medicine approach to the management of lung cancer.

## Introduction

### Lung Cancer

Lung cancer is the leading cause of cancer related deaths and accounted for over 2.1 million new cases and 1.8 million deaths in 2018 (1). If the cancer is diagnosed in the early stages (i.e., Stage I – II), the five-year survival rate is estimated to be around 56% (2). However, only 16% of lung cancer cases are diagnosed early, which causes the overall five-year survival rate of lung cancer patients to be less than 20% with adverse clinical outcomes (3, 4). Lung cancer is mainly categorized into two histological groups: non-small cell carcinoma (NSCLC) and small cell lung carcinoma (SCLC) (5). NSCLC is the most prevalent lung cancer type that accounts for 80% to 85% of all lung cancer cases and is further divided into three histological subtypes of adenocarcinoma, squamous cell carcinoma, and large cell (undifferentiated) carcinoma (6). Adenocarcinoma accounts for 40% of all lung cancers and is frequently found in peripheral bronchi (7, 8). Squamous cell carcinoma comprises 25%-30% of all lung cancer cases which arises from the main bronchi and disseminates into the carina. Large cell (undifferentiated) carcinoma represents about 10% and may originate from different part of the lung. SCLC accounts for 10-15% of all lung cancers and it is the most aggressive type with the lowest overall survival (OS) (5, 6).

## Liquid Biopsy

Lung cancer patients often suffer from progression of their disease with adverse clinical outcomes, complications and recurrence (9). Therefore, early diagnosis is vital for effective disease management and preventing the advancement of cancer. However, one of the main challenges for managing and treating lung cancer patients is the lack of sensitive early diagnostic methods (10). To date, low-dose spiral computed tomography (LDCT) is the most commonly used approach for lung cancer screening with more than four times higher sensitivity compared to X-ray imaging (3, 11, 12). However, high false-positive results in the early stages of lung cancers and radiation exposure often limits the usage of LDCT (10).

Tumour biopsy is the gold standard for lung cancer diagnosis due to the potential of investigating targeted biomarkers including carcinoembryonic antigen (CEA), fragments of cytokeratin ‐19 (CYFRA21‐1), squamous cell carcinoma antigen (SCC) and neuron‐specific enolase (NSE) (13, 14). However, tumour biopsy is an invasive approach, requiring specialist medical expertise and cannot be routinely performed outside of a hospital setting. Single site biopsy may have a sampling bias by not being representative of the whole tumour (15). Moreover, tumour biopsies represent a single time point snapshot of the tumour, where therapy-induced changes cannot be determined over the course of treatment. Therefore, alternative, more dynamic biomarkers which can be assessed serially over the course of therapy are desirable (14).

**Figure 1:**
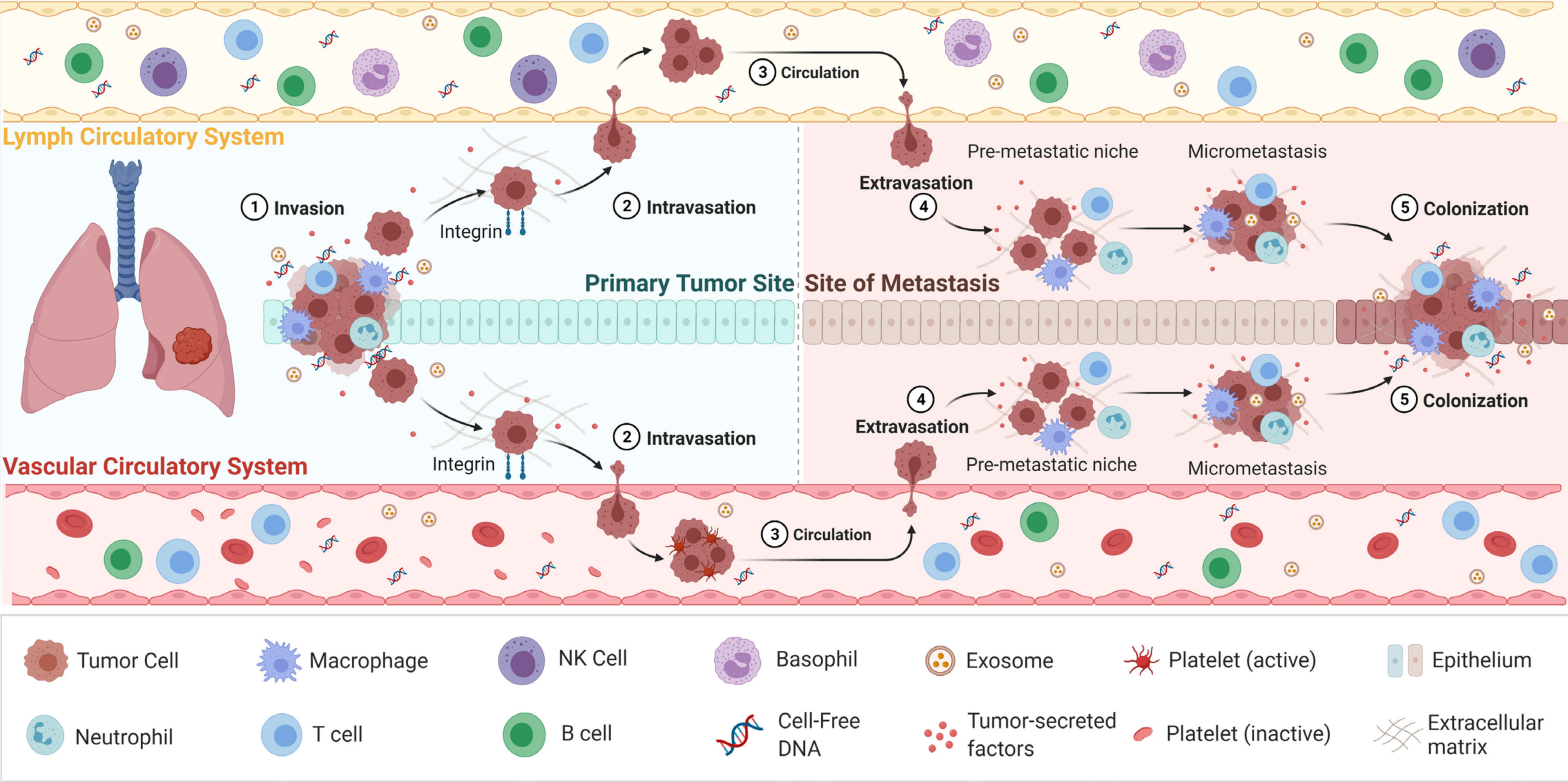
The process of cancer metastasis. Metastasis occurs *via* the vascular or lymph circulatory systems, where cancer cells from the primary tumour, intravasate into the blood/lymphatics systems and travel through the body, and extravasate at local/distant sites/organs. Adapted from “Overview of Metastatic Cascade”, by BioRender.com (2021). Retrieved from https://app.biorender.com/biorender-templates.

**Figure 2:**
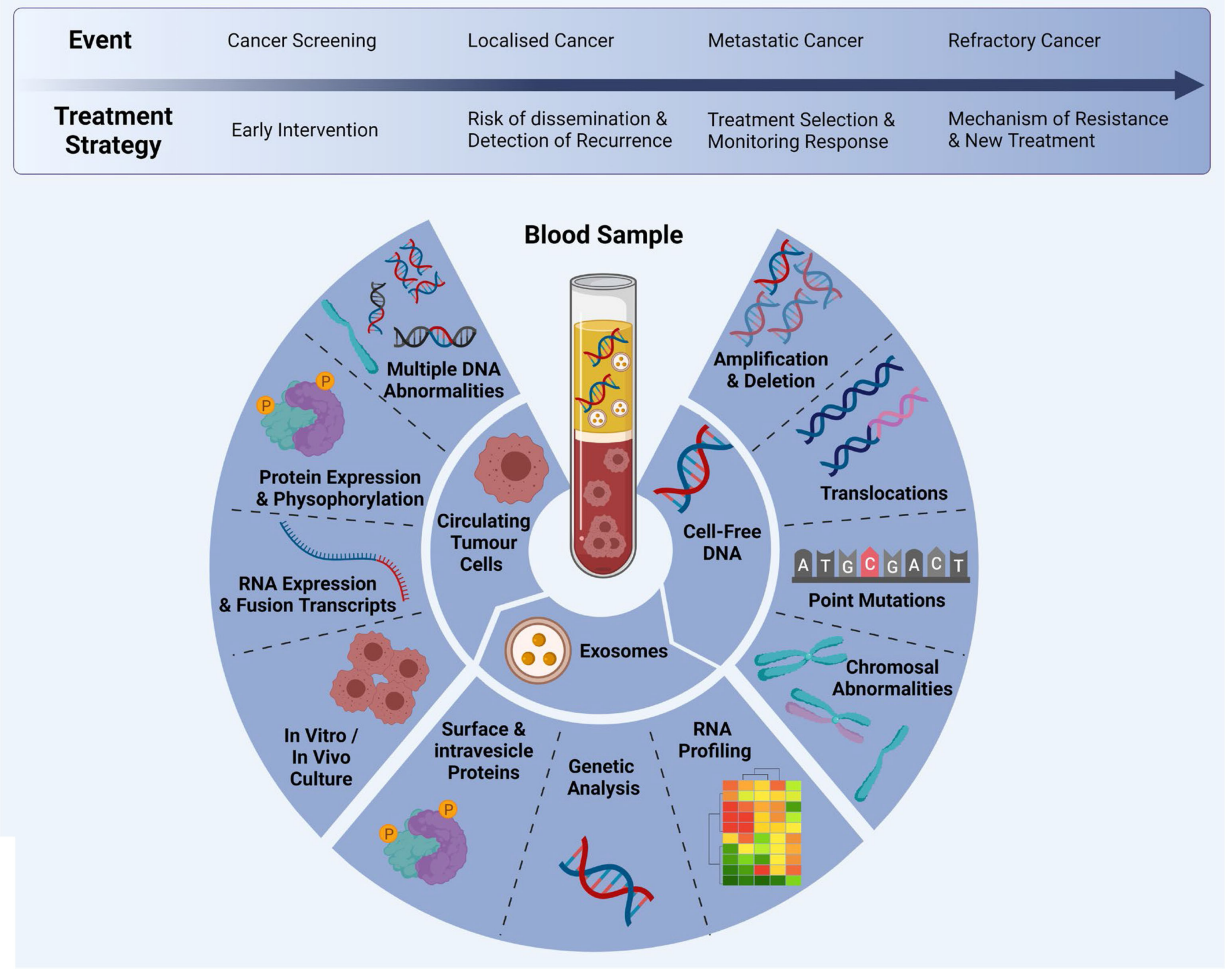
Multi-marker analytes in a liquid biopsy sample: CTCs, cfDNA and exosomes can be analysed at different time points during clinical progression/treatment. Adapted from Ref (20) and created with BioRender.com.

Liquid biopsy have emerged as a promising tool to detect tumour biomarkers (**Figure 1**) in body fluids in a non-invasive manner. They have shown to play a crucial role in lung cancer screening, early diagnosis, monitoring and determining patient prognosis (16). The minimal invasiveness, ease of use and ability to have repeat measurements over time make it a useful companion diagnostic tool. To date, liquid biopsies has been developed as a novel diagnostic approach to explore potential circulating tumour biomarkers and is considered as a reliable way to understand dynamically changing tumour biology under the stressors of treatment. Liquid biopsy biomarkers include cell-free DNA (cfDNA), circulating tumour DNA (ctDNA), microRNA (miRNA), exosomes and circulating tumour cells (CTCs) (3, 12, 17–19). This review provides an overview of these circulating tumour biomarkers and their clinical significance in screening, early diagnosis and prognostication of lung cancer (**Figure 2**).

### cfDNA and ctDNA

Cell free DNA (cfDNA) is derived from dividing and apoptotic cells as a result of the normal physiological process of tissue remodeling (21–23). In healthy subjects, the amount of cfDNA is quite low and it has been estimated as 5–10 ng/mL in body fluids (24). The half-life of cfDNA is approximately 2 hours (25). The proportion of cell free DNA derived from tumour cells is known as ctDNA (18). The concentration of ctDNA in plasma varies from 0.01% to 90% of total cfDNA (25). The amount of ctDNA present in plasma correlates with the tumour burden, progression free survival (PFS) and OS (10), which is a useful biomarker to monitor NSCLC patients in different stages (26). A study conducted by Newman et al. has been found that a 100% detection rate of ctDNA in stage II–IV NCSLC patients and 50% in early-stage patients (26). Owing to the short half-life of ctDNA, treatment efficacy of patients can be rapidly detected, earlier than radiological changes (25). On the other hand, in order to detect ctDNA in the peripheral circulation, blood samples should be collected within a certain time period to avoid degradation (24). ctDNA can identify specific molecular changes present in the original tumour. These include mutations in oncogenes/tumour suppressor genes and gene amplifications or epigenetic changes (19). Therefore, cfDNA/ctDNA has been used as a prognostic marker for the diagnosis of different types of cancers including lung cancer (17, 27, 28). Furthermore, ctDNA provides a molecular picture of the residual disease which helps in decision making for the commencement of adjuvant chemotherapy after the surgery (29). The concentration of cfDNA is found to be higher in early stage NSCLC patients, recurrent and advanced stage NSCLC patients compared to healthy subjects (26, 29). A study by Pohomaryova et al., reported that the plasma concentration of cfDNA in lung cancer patients is eight times higher than normal healthy adults (30). Several studies have been reported the presence of high cfDNA concentrations has a significant association with the worse clinical outcome (31–34). Furthermore, cfDNA plays an important role for identifying blood tumour mutational burden (bTMB) in NSCLC, indicating the number of somatic mutations in the genome coding regions (35). It has been found that sensitivity and specificity of bTMB assay were 93.9%, 93.9% and 100.0%, compared to tissue TMB (36).

cfDNA mutation analysis is also important to identify specific mutations and molecular targets for personalized therapies (37). This can be beneficial to select patients for immunotherapy and pursue a clinically meaningful improvement in terms of survival (38, 39). The United States Food and Drug Administration (U.S. FDA) approved ctDNA as the first liquid biopsy test for the detection of NSCLC patients with EGFR mutations who were suitable for personalized therapy (Roche Cobas EGFR mutation test v2) (40). This assay can detect multiple mutations in exons 18, 19, 20 and 21 in NSCLC including L858R, T790M, G719X, S7681, and L861Q (41). Using ctDNA, 62.5% of patients were identified with EGFR mutations (exon 19 deletion, exon 20 T790 M insertion and exon 21 L858R mutation) at the baseline, while the rate of EGFR mutation positivity was higher among patients with metastatic disease (42). ctDNA, as a specific tumour marker, has a high specificity of 80-95% for the detection of EGFR mutations, which can inform on the use of tyrosine kinase targeted therapies. However, the sensitivity of this approach is comparatively lower – about 60-85%, which cannot be used to ensure the EGFR mutation does not exist in the patient. In addition to detection of EGFR mutation, ALK rearrangements have been assessed in ctDNA of lung cancer patients (43–45). ALK is a membrane-bound tyrosine kinase receptor encoded by the ALK gene (46, 47) and rearrangement of ALK can be seen in 2-7% of NSCLC patients. Hence, ALK rearrangement has emerged as the second most studied targetable mutation in order to develop a novel treatment approach to lung cancer (48).

In addition to single-gene assays, higher through-put NGS multi-gene readout assays have been developed to identify single-nucleotide variants (SNVs), copy number alterations (CNAs), inserts/deletions and or fusions. These assays include the FoundationOne Liquid CDx, Guardant 360 CDx, MSK-ACCESS, OncoDNA and Archer Reveal. These assays have the advantage of broad genotyping and utility in clinical trials (49). The blood TMB (bTMB) assays have shown concordance with the tumour TMB assays in advanced stage NSCLC, in particular for predicting clinical outcomes to immunotherapy (50, 51).

Usually, at the time of sample collection, tumour-derived DNA is found highly fragmented and mixed with non-tumour DNA/cfDNA. Separation of ctDNA from cfDNA can be pursued using ultrasensitive analytical assays (52). Several testing approaches have been identified; including emulsions, beads, amplification, magnetics (BEAMing), droplet digital PCR (ddPCR) and next generation sequencing (NGS), which can detect down to a few copies of ctDNA (53–55). In contrast, the low amount of ctDNA in plasma has been challenging the clinical applicability of this technique. Therefore, evaluation of ctDNA in other body fluids than plasma may potentially provide a solution to this. The concentration of cfDNA in body fluids is higher near the proximal tumour sites (37). It is worth mentioning that the current guidelines emphasize plasma as a preferred specimen than serum for the detection of ctDNA, as leukocyte lysis takes place during clotting which thereby leads to high contamination rate of germinal DNA than in plasma (56). Furthermore, the specimen should be processed within 6 hours of collection in order to prevent release of DNA from normal blood cell lysis and leukocyte stabilization reagents would be useful in these circumstances (57).

### Exosomes

Exosomes are small extracellular vesicles derived by endocytosis, with 40–100 nm in size (58), which can be found in all the body fluids and capable of transporting DNA, mRNAs, noncoding miRNAs, proteins and lipids (59). It consists of a lipid bilayer that prevents degradation by enzymes such as ribonuclease and high pH conditions. Hence, exosomes play an important role in cell mediated communication in normal healthy cells and disease cells including tumour cells (60). All cell types, including normal, disease or tumour cells can release exosomes into the extracellular space (61). Recent studies suggest a higher number of exosomes in patients with cancer than in healthy adults (62, 63). There is emerging evidence that exosomes have a crucial role in carcinogenesis, cancer progression and metastasis of several tumours, including NSCLC (14).

Exosomes derived from tumour cells can promote carcinogenesis by transferring oncogenic factors and thereby induce malignant transformation (64). Acquiring the oncogenic factors from by non-cancerous cells leads to change of cellular behaviour and share comparable characteristics that the tumour poses (65). Tumour derived exosomes also have the potential of influencing epithelial-mesenchymal transition (EMT) and facilitate metastasis by transferring migratory and metastatic capacity to the non-cancerous cells (66). Moreover, these extracellular vesicles cause the expression of vimentin in normal cells, stimulating EMT and increasing the metastasis ability. This can further facilitate neoangiogenesis by releasing proteins such as fibroblast growth factor (FGF), vascular endothelial growth factor (VEGF), IL-6, and IL-8, and stimulating vascular endothelial cells *via* miRNAs (67, 68).

The analysis of nucleic acids in exosomes has shown to be a more reliable and sensitive mutation detection approach than using cfDNA/ctDNA. Thakur et al. showed tumour-derived exosomes carry double-stranded DNA which represents the whole genome and the mutation profile of the primary tumour cells (69). Exosomes hold great promise as biomarkers for the early diagnosis and treatment *via* analysis of nucleic acids and other markers to find clues on primary tumour and metastasis condition of patients. Different exosomal proteins and miRNA have been currently employed for the diagnostic and prognostic utility. Some surface proteins offer promising potential as tumour markers including CD91, CD317 and EGFR (70).

Exosomal microRNA-96 (miRNA-96) fosters lung cancer progression by suppressing the activity of Lim domain 7 (LMO7) protein. LMO7 is a fibrous actin-binding protein functions in the formation and maintenance of actin cytoskeleton. In lung cancers, it functions as a tumour suppressor molecule while a shortage of LMO7 confers a genetic predisposition to lung cancer (67). Studies have been documented that the amount of exosomal miRNA is different between healthy individuals and lung cancer patients at different stages (71, 72). Therefore, exosomal miRNA is imperative as a promising and effective noninvasive candidate biomarker for early diagnosis, tumour profiling and enabling a timely treatment plan in NSCLC (10).

It has been found that EGFR protein can be transferred from tumour cells to non-cancerous cells *via* exosomes which leading to downregulation of the VEGF pathway (73). Similar to cfDNA, exosomal RNA could be utilized to detect EGFR mutations. It is worth mentioning that exosomal RNA has been found to be more efficient in analyzing EGFR mutations than cfDNA (74).

Although exosomes have provided prospective clinical evidence on cancer, still several constraints limit their clinical utility. One of the major drawbacks is difficulty in purification due to its’ smaller size. Further, few drawbacks are associated with the difficulty to adopt exosome isolation techniques in clinical environment. In this context, several isolation techniques have been employed based on the physical, chemical and biological properties of exosomes (75). Physical methods include ultracentrifugation, ultrafiltration, density gradient separation and size exclusion chromatography (75). All these aforementioned methods are based on the molecular size and density of the exosomes. In the ultracentrifugation, according to the density, blood and other components are separated with different centrifugal speeds and exosomes are separated in the final ultracentrifugation step. Ultrafiltration is another physical method that utilizes the exosome size for separation of these extracellular vesicles (59). Furthermore, Polymeric-based precipitation is one of the promising techniques that used the chemical properties of exosomes by using beads coated with specific antibodies coated.

### CTCs

CTCs were first described by an Australian physician, Thomas Ashworth in 1869, where he observed cells similar to the primary tumour were found in the blood of a patient with metastatic disease. Accordingly, metastasis is thought to be facilitated by metastatic precursor cells, known as CTCs, which are tumour cells that detach from a tumour into the vasculature (76). They are extremely rare and approximately 1-10 CTCs can be present per milliliter of whole blood (77). Emerging evidence has demonstrated that the presence of CTCs in the vascular system associate with worse clinical outcomes in terms of OS of cancers (5). It has been shown that patients with NSCLC have ten times higher concentration of CTCs in their blood circulation than in other cancer patients (78–80).

CTCs are considered to play a pivotal role in metastasis of cancer and during which they go through a process called EMT (81, 82). EMT is a complex biological process that results in the gradual lowering of epithelial features of the tumour cells and obtains mesenchymal properties to acquire migratory and invasive metastatic characteristics (83). In this process, tumour cells enter the circulation and reach a distant site where a metastatic deposit is initiated. Here, CTCs regain epithelial characteristics allowing further proliferation and formation of metastases deposits at the distant site (**Figure 1**) (84). A sensitive and specific isolation method is therefore imperative to provide sufficient and purified CTCs to analyse (85, 86). Since CTCs are extremely rare, their isolation is greatly constrained by technological implications (87, 88). With the advancement of novel technologies for CTC enrichment, in particular that of microfluidics, CTC capture is becoming increasingly efficient. The current CTC enrichment methods are based either on the biological or physical properties of CTCs. The CellSearch (Menarini Silicon Biosystems) platform is the only FDA-approved CTC enumeration method which target epithelial cell adhesion molecule (EpCAM), a transmembrane glycoprotein involved in cell to cell adhesion (89). This platform is approved for breast, colon and prostate cancers. However, this platform has a number of limitations, including that is only captured epithelial CTCs and does not capture mesenchymal shifted CTCs with low or absent EpCAM expression (90, 91). Furthermore, isolation and enrichment of CTCs have been extremely challenging in NSCLC due to the downregulation of the epithelial tumour markers during EMT, which consequently masks the true number of CTCs (10). CTCs often have partial-EMT or hybrid states, which can downregulated markers such as EpCAM (92–94). This is further confounded by the presence of CTC clusters or microemboli which have a higher metastatic capacity compared to single CTCs, including immune evasion strategies by the inclusion of leukocytes (83, 95–97).

To tackle these drawbacks, label-free CTC isolation technologies have been established to isolate CTCs based on size, density and deformability (87, 98). Still, considerable constraints of label-free technologies are inevitable and the usage is limited due to their throughput (99). To overcome this, recently microfluidic technologies have been developed including electrophoresis, hydrodynamic and cross-flow filtration, micropore and micropost trapping, deterministic lateral displacement and inertial focusing systems to capture CTCs (90, 99–103). This has added values through effective cell sorting without need for purification, high system throughputs and ability to analyze functions of CTCs *in vitro* (90, 99, 104–106).

Isolated CTCs can be analysed through different approaches including molecular and proteomic studies. For instance, captured CTCs can be used for EGFR mutation analysis which will provide a better understanding of the tumour genetic profile similar to ctDNA (10). Studies emphasized that detection of EGFR mutations in CTC could assist in determining prospective therapeutic decisions which ultimately would lead to the advancement of precision medicine and personalized oncology (5, 107). Other than EGFR mutations, programmed death ligand-1 (PD-L1) expression on CTC has also been studied; however, authors have not been established a clear relationship between its expression and cancer progression and prognosis in patients with NSCLC (108, 109).

The isolation of a sufficient amount of CTCs in the blood of NSCLC patients and identify its various biomarkers can aid in early detection of the NSCLC and will also provide real-time monitoring of cancer progression, treatment efficacy and prognosis.

### Circulating miRNAs

miRNAs are a type of gene expression regulator that works at the post-translational level with a multi-protein complex known as the RNA-induced silencing complex (miRISC), exerting their function at the 3’-untranslated region (3’UTR) of target complementary messenger RNA (mRNA) sequences (110, 111). miRNAs have been discovered to be single-stranded RNA molecules with a length of 19 to 22 nucleotides (14, 112). It has been well established that each miRNA can regulate and act as a target for multiple mRNAs and that each mRNA, resulting in a cascade of gene regulation (113, 114). Evidence suggests that any dysregulation of miRNAs could have an impact on a variety of diseases, most notably cancer (115). In the context of cancer, miRNA dysregulation has been linked to tumor initiation, growth, and progression, with evidence pointing to miRNAs acting as tumor suppressors and oncogenes (115). In addition, it was found that miRNAs are packed into extracellular vesicles before being released into the extracellular space. These vesicles could be exosome and microvesicles (116–118). Several studies have been conducted to explore the role and application of miRNAs in cancer (75, 119–121). The serum or plasma level of miRNAs can serve as a predictive marker in cancer patients, indicating signs of the disease stage (75, 119). miRNAs are released into the circulation *via* protein-miRNA complexes, exosomes, tumor-educated platelets (TEP), and apoptotic bodies (122, 123). Accordingly, evidence suggests that cancer therapeutic approaches such as chemotherapy and radiotherapy may affect circulating miRNA levels, implying that miRNAs can serve as biomarkers for response or resistance to therapy (120, 121, 124, 125). In lung cancer, miRNAs have been found to distinguish healthy individuals from cancer patients (126–128). miRNAs may also serve as biomarkers of therapeutic success in NSCLC patients, allowing for better patient management decisions (129). As a result, studies on lung cancer patients revealed that, when compared to LDCT, miRNAs had significantly lower false-positive results when it came to detecting lung cancer patients in their early stages (129). Furthermore, a meta-analysis study from 65 studies (6919 patients with lung cancer and 7064 healthy volunteers), discovered a panel of four miRNAs, including miR-21-5p, miR-126-3p, miR-155-5p, and miR-223-3p, which can be used as a potential biomarker in lung cancer screening (130). Moreover, a different study conducted on early NSCLC patients showed that a number of miRNAs were differentially regulated between short-term survivors and long-term survivors. These miRNAs, which included miR-1, miR-30d, miR-486, and miR-499, were also linked to the OS (127). In line with these findings, Li et al. studied miR-486 and miR-150 on plasma samples from lung cancer patients to explore their early diagnostic value (131). As a result, these miRNAs were discovered to have greater than 80% specificity and sensitivity in discriminating healthy individuals from lung cancer patients (131). Another study on patients with metastatic lung cancer discovered that miR-18a, miR-28-3p, miR-191, miR-145, and miR-328 were associated with 3-year survival (132). In studies on NSCLC tumors, researchers discovered six serum miRNAs from patients that had significantly different expressions when compared to healthy people. Accordingly, miR-15b-5p was found to be overexpressed, while miR-19-3p, miR-92-3p, miR-16-5p, miR-17b-5p, and miR-20a-5p were found to have downregulation (133). Among these miRNAs, miR-16-5p, miR-15b-5p, and miR-20a-5p had the highest sensitivity and specificity values (133). However, despite these promising findings, there are some drawbacks to using miRNAs as diagnostic biomarkers, which are listed in **Table 1**.

**Table 1:** Comparison of different liquid biopsy markers including cfDNA/ctDNA, exosome, CTC, cmiRNA and TEP.

Marker	Sample type	Strength	Weakness	Clinical application in oncology	Ref.
cfDNA/ctDNA	Serum Plasma CSF Ascites Pleural effusion	Reflective of tumor molecular alterations/mutations Stable up to 2 days in blood samples Reflective of tumor heterogeneity Highly sensitive assays (NGS, PCR)	Contamination of germinal cfDNA Cannot reflect every gene mutation Low amount in plasma Undetectable in many patients with early-stage cancer Less stable than non-tumor DNA	Elevated in cancer patients compared to healthy individuals Increases with tumor size and stage	(14, 49, 134, 135)
Exosomes	Nearly all body fluids	Stable source of tumor genetic material (DNA, RNA, protein, miRNA) Commercial kits available	required standardization for extraction and detection Unreliable isolation procedures	Elevated in cancer patients compared to healthy individuals Exosome size positively correlates with unfavorable outcomes	(5, 110, 136)
CTC	Peripheral blood	Assessment of tumor markers (PD-L1) during treatment Demonstration of signal co-localization Cell morphology and functional studies	Not predictive of therapeutic benefit in metastatic setting Undetectable in most patients with early-stage cancer Rare to capture in the bloodstream	Predictive of early relapse after primary treatment CTC number correlates with progression-free survival and overall survival	(109, 122, 137)
cmiRNA	Serum Plasma	Different profile among early-stage cancer patients Distinguishable between cancer patients and healthy individuals	Loss of epithelial specific markers during epithelial mesenchymal transition (EMT) High variability Lack of standardization Unspecific for a cancer type	cmi-RNA expression correlates with tumor development, progression and metastasis	(14, 110, 122)
TEP	Peripheral blood	TEP-RNA is reflective of tumor transcriptome Abundant Dynamic mRNA repertoire because of short life-span	Reproducibility Lack of validated assay	Distinguishable between healthy individuals and cancer patients Distinguishable between patients with early-stage cancer and patients with advanced-stage cancer	(138, 139)

The sample type, strength, weakness and clinical applications of each marker is discussed.

### Tumor Educated Platelets

Platelets are enucleated cells that participate in coagulation. Platelet activation has also been found in inflammatory diseases like eczema and asthma (140). Evidence suggests that platelets may play an important role in tumorigenesis by helping tumor evasion, angiogenesis, and metastasis (141, 142). Activated platelets secrete α-granules to release transforming growth factor-beta (TGFβ) and adenosine triphosphate (ATP), leading to epithelial mesenchymal transition (EMT) and metastasis (143). On the other hand, tumour cells have been found to induce thrombocytosis by producing growth factors and cytokines such as granulocyte colony-stimulating factor (G-CSF), granulocyte-macrophage colony-stimulating factor (GM-CSF), interleukin (IL)-1 and IL-6 (144). Given this information, the interaction between tumour, tumour microenvironment (TME), and platelets account for tumor-educated platelets (TEPs) (145, 146). Accordingly, numerous studies explored the use of TEPs as a liquid biopsy in cancer initiation and progression (147–150). It has been discovered that growth factors released and produced by platelets and tumor cells, such as vascular endothelial growth factor (VEGF), contribute to changes in mRNA expression within platelets, resulting in a specific spliced mRNA signature (151, 152). The mRNA signature could be used as a biomarker to distinguish cancer patients from healthy individuals. According to multiple studies, patients with cancer onset and progression have a highly dynamic mRNA repertoire (138, 153). Therefore, analyzing mRNA profiles may be useful in detecting primary tumors, metastasis, and cancer staging (45, 138). A study that compared patients with localized and metastatic tumors to healthy people discovered that platelet mRNA profiles could distinguish cancer cases from healthy ones with 96% accuracy, 97% sensitivity, and 94% specificity (153). In the context of lung cancer, Geraci and colleagues investigated platelet mRNA profiles in the context of lung cancer using a lung cancer model. As a result, they discovered distinct platelet mRNA gene expression between metastatic and control groups (154). Furthermore, research has revealed that some patients with lung adenocarcinoma have ALK rearrangement, resulting in the EML4–ALK fusion gene product (155, 156). Using reverse transcription-polymerase chain reaction (RT-PCR), Nelson et al. examined blood platelets from 77 NSCLC patients and found 38 cases had an EML4-ALK-rearrangement in platelets with 65% sensitivity and 100% specificity (157). In addition, it was reported that the EML4-ALK rearrangement in platelets had a correlation with PFS and OS in patients who received crizotinib. Accordingly, patients with EML4-ALK+platelets had 3.7 month PFS, whereas patients with EML4-ALK- platelets had 16 month PFS (157). When compared to patients with non-cancerous inflammatory diseases, patients with cancer have a hyperactive state of TEPs, according to functional analysis (158). In addition, using RNA sequencing, the platelet RNAs were investigated in patients with early stage NSCLC and healthy individuals. It was demonstrated that integrin alpha-IIb (ITGA2B) was expressed more in NSCLC patients than healthy individuals (159). As a result of their findings, the researchers concluded that TEP ITGA2B could be a promising marker for detecting patients with early-stage NSCLC (159).

## Discussion

**Figure 3:**
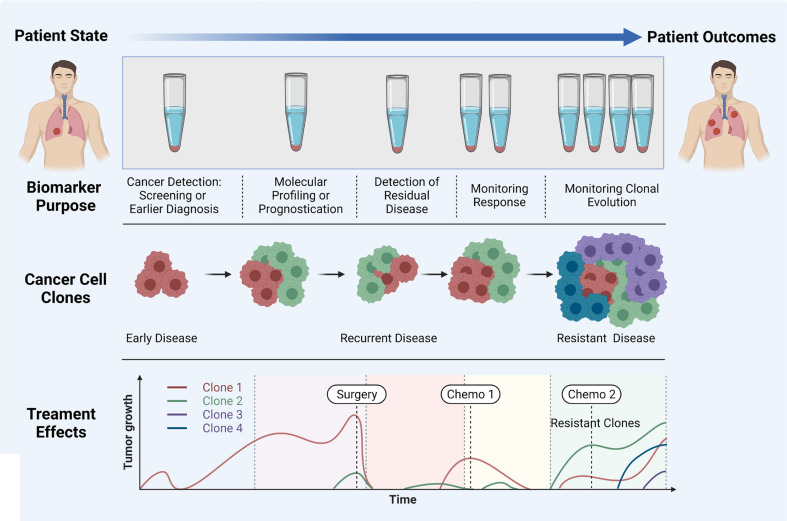
Importance of liquid biopsy at different stages of cancer propagation, indicating the biomarker purpose, evolution of clones, and treatment effects. Adapted from Ref (160) and created with BioRender.com.

Liquid biopsy has shown promise for the early diagnosis and management of lung cancer due to its high sensitivity, specificity, non-invasive sampling and low-risk profile. cfDNA/ctDNA, CTCs, miRNAs and exosomes are considered as potentially actionable biomarkers which are used in liquid biopsy. However, robust characterization of each marker is needed for the comprehensive understanding of their role in NSCLC disease progression, prognostication and determining a tailored treatment regimen. It is clear that certain liquid biopsy analytes would be useful over the course of disease progression. For example, ctDNA and exosomes may be informative early in disease onset, and could be used to identify specific actionable mutations/and early stage disease which cannot be determined using LDCT. Whereas CTCs may be used to identify the risk of developing metastatic disease, and the types of clones which may develop treatment resistance (**Figure 3**). ctDNA has also shown utility over the course of therapy where the variant allele frequencies can be monitored over time/therapy to determine increases or decreases of tumour specific mutations in response to treatment. ctDNA has been the most studied marker in the field of lung cancer with clinical utility for a number of gene mutations. The detection of tumour specific mutations post therapy may also present a window of therapeutic opportunity where the patient has minimal residual disease (MRD), prior to clinically detectable disease progression with radiological evidence. Whilst larger multi-marker NGS panels have come onto the marker, cross comparisons of blood and tumour studies will be warranted to determine their utility for targeted therapies. Moreover, large prospective clinical trials are needed to provide a better understanding of the clinical utility of liquid biopsy assays.

## Author Contributions

All authors listed have made a substantial, direct, and intellectual contribution to the work, and approved it for publication.

## Funding

The authors are supported by project grants and fellowships for AK from NHMRC (1157741), Cure Cancer (1182179), and the PA Research Foundation (KOB).

## Conflict of Interest

The authors declare that the research was conducted in the absence of any commercial or financial relationships that could be construed as a potential conflict of interest.

## Publisher’s Note

All claims expressed in this article are solely those of the authors and do not necessarily represent those of their affiliated organizations, or those of the publisher, the editors and the reviewers. Any product that may be evaluated in this article, or claim that may be made by its manufacturer, is not guaranteed or endorsed by the publisher.
